# Is EGFR expression altered following postoperative chemotherapy for colorectal adenocarcinoma?

**DOI:** 10.1186/1477-7819-4-92

**Published:** 2006-12-12

**Authors:** Mahmoud A Khalifa, Corwyn H Rowsell, Rebecca Gladdy, Yoo-Joung Ko, Sherif Hanna, Andy Smith, Calvin Law

**Affiliations:** 1 Department of Pathology, Sunnybrook Health Sciences Center, Toronto, Canada; 2 Surgical Oncology, Sunnybrook Health Sciences Center, Toronto, Canada; 3 Medical Oncology, Sunnybrook Health Sciences Center, Toronto, Canada

## Abstract

**Background:**

There is immunohistochemical evidence to suggest that expression of epidermal growth factor receptor (EGFR) in primary colorectal adenocarcinoma predicts its expression in recurrent disease. This study investigates whether postoperative chemotherapy affects the degree of concordance between EGFR statuses of the two tumors.

**Methods:**

Thirty-three patients were identified from the files of Sunnybrook Health Sciences Center from July 1994 to June 2005. All patients had resection of their primary tumors and their distant recurrences. Eighteen patients received postoperative chemotherapy, 3 of which also received postoperative radiation therapy. Representative primary and recurrent tumor sections were stained using mouse anti-EGFR antibodies and only membranous staining of malignant cells was recorded. Results were reported as negative (no staining), 1+ (positivity in <50% of cells) or 2+ (positivity in >50% of cells).

**Results:**

EGFR immunostaining in the 15 patients, who received no postoperative chemotherapy, was decreased in 3 recurrences, remained the same in 10 and increased in 2. In the group of 18 patients who received postoperative chemotherapy, EGFR immunostaining was decreased in 6 recurrences, remained the same in 9 and increased in 3 (p = 0.6598). In patients who received postoperative chemotherapy, the odds ratio for a recurrence to show lower levels of EGFR immunostaining compared to its originally resected primary was 4.75 (CI = 0.94 – 26.73).

**Conclusion:**

These preliminary data suggest that recurrences following postoperative chemotherapy are likely to have lower levels of EGFR expression compared to cases who receive no chemotherapy. Although the difference of immunostaining profiles between the two groups was not statistically significant, this observation might impact the management of these patients by targeted biologic therapies and its practical implications need further validation in larger series.

## Background

Epidermal growth factor receptor (EGFR) is a transmembrane glycoprotein, which is reported to be overexpressed in approximately 70 to 75% of colorectal cancer [1]. It consists of an extracellular ligand-binding domain, a transmembrane region, and an intracellular tyrosine kinase domain [2,3]. Its signaling pathways have been linked to tumor proliferation, invasion, cellular migration, angiogenesis, and resistance to apoptosis [4]. Cetuximab (Erbitux) is a human/murine chimeric antibody (IgG1) that targets the extracellular domain of EGFR with high specificity and affinity [5]. Clinical trials in the setting of metastatic colon cancer refractory to chemotherapy have shown efficacy with modest toxicity, both as a single agent and in combination with irinotecan [6-8].

The US Food and Drug Administration approved Cetuximab in February 2004 for use as a third-line therapy in patients with metastatic colorectal cancer refractory to irinotecan. Studies in patients with EGFR expressing tumors demonstrated a superior response rate and longer time to progression in those who received Cetuximab [8]. In these studies, EGFR status was mostly determined by immunohistochemical staining of primary tumor samples rather than from recurrent or metastatic tumors [6-8]. There is established immunohistochemical evidence to suggest that EGFR expression in primary colorectal adenocarcinoma predicts its expression in recurrent disease [9-11]. However, the potential changes in the tumor's EGFR status due to postoperative chemotherapy effects have not been addressed. The question of whether modulation of EGFR status by chemotherapy can occur has already been address in other tumors [12]. Induction chemotherapy has been shown to induce EGFR expression in are cases of EGFR-negative non-small cell lung cancer. This study investigates whether postoperative adjuvant therapy affects the degree of concordance between EGFR statuses of the primary and recurrent colorectal cancer.

## Materials and methods

### Specimen selection

During the period of July 1994 – June 2005, 33 colorectal adenocarcinoma patients with distant recurrence were captured in our pathology database. Surgical resection of all primary tumors was performed. Eighteen patients received postoperative chemotherapy; three of which also received postoperative radiation therapy. All resected metastases were metachronous. Liver segmentectomy or lobectomy was undertaken at a later time for 31 patients and lung lobectomy for 2 patients to resect their distant recurrences. All hematoxylin and eosin-stained slides from the resected primary and recurrent tumors were retrospectively reviewed to confirm the diagnosis and to select a block for immunohistochemical staining.

### Immunohistochemistry

According to our local protocols, all resected tissue specimens were fixed for 24 hours in 10% neutral buffered formalin. At the time of the study, the selected paraffin blocks had been stored for 7 – 122 months (mean = 51, ± 36). Immunostaining was performed on 5-μm-thick formalin-fixed, paraffin-embedded tissue sections, using a Dako Autostainer (DAKO, Carpinteria, CA) according to the manufacture's specifications. Sections were stained using mouse anti-EGFR antibodies (Zymed Laboratories, Inc., San Francisco, CA). The antibody used was clone 31G7 and sections were incubated for 30 minutes at room temperature at the dilution of 1:100. The antibody manufactured by Zymed Laboratories was chosen for the current study since this was the antibody employed in our local laboratory for the past 3 years and since it was one of the three antibodies equally recommended by the Canadian Consensus Panel on EGFR Testing in Colorectal Cancer [13].

Predigestion by pepsin at 37°C for 10 minutes was performed. Positive (ductal carcinoma of the breast) and negative controls were stained with every run. To avoid any potential interference by endogenous biotin in liver tissue, the biotin-free detection system, mouse-probe HRP polymer kit (MACH 3™) by Biocare Medical (Walnut Creek, CA) was used. Sections were immunostained in batches and were all processed by a single experienced immuno-histotechnologist.

### Evaluation and analysis

**Figure 1:**
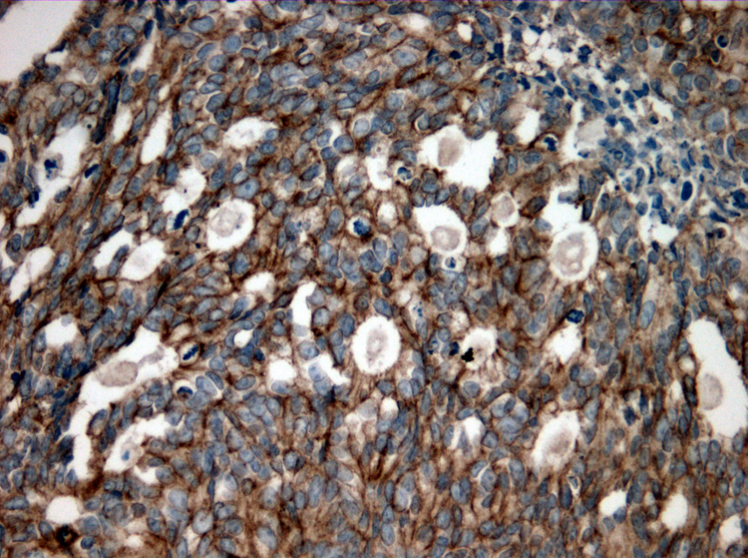
EGFR immunopositivity in > 50% of cells in a metastatic colorectal adenocarcinoma to the liver (original magnification × 200).

Positive staining was defined as any membranous brown staining of malignant cells above background level (Figure 1). Cytoplasmic staining without associated membrane staining was considered as negative. The immunostaining results were recorded on a three-tier scale as negative (no staining), 1+ (positivity in <50% of cells) and 2+ (positivity in >50% of cells). In order to augment objectivity of our results, intensity of staining was not included as a reportable variable. Immunostaining of each of the primary and recurrent tumors was assessed blindly, without knowledge of the immuno-status of its counterpart. The clinical data were obtained from the patient's electronic charts. SAS 8.2 (SAS Institute, Cary, NC) system was used for data analysis.

## Results

Patients ranged in age at the time of primary diagnosis from 41 to 80 years old (mean = 65.4 ± 10.2). Their clinical data are summarized in Table 1. One patient with stage I disease and 2 with stage II were suspected to have local recurrence shortly after resection of their primary tumors for which they received chemotherapy. Five patients with stage III and one with stage IV did not receive adjuvant chemotherapy for a variety of comorbidity reasons. None of the patients had received EGFR-targeted therapy.

**Table 1:** Clinical characteristics

**Characteristic**	**Number of patients**	
	
	Postoperative chemotherapy	No postoperative chemotherapy
Sex		
Males	11	9
Females	7	6
Primary tumor location		
Cecum	3	2
Colon	2	2
Sigmoid colon	7	6
Rectum	6	5
Tumor stage at the time of diagnosis		
Stage I	1	0
Stage II	3	9
Stage III	14	5
Stage IV	0	1
Surgery		
Right hemicolectomy	3	3
Anterior resection	1	3
Low anterior resection	9	2
Subtotal colectomy	1	0
Pelvic exenteration	0	5
Left hemicolectomy	1	0
Abdomino-perineal resection	2	2
Total proctocolectomy	1	0
Additional therapy		
Neoadjuvant chemotherapy	1*	3*
Neoadjuvant radiation therapy	0	6
Postoperative chemotherapy	18	0
Postoperative radiation therapy	3**	0

* Also received neoadjuvant radiation therapy

** Also received postoperative chemotherapy

As shown in Table 2, twenty-one (63.6%) of the primary tumors and 17 (51.5%) of their distant recurrences had positive EGFR immunostaining. Nineteen of the 33 (57.6%) cases showed the exact degree of immunopositivity in both primary and recurrent tumors. As shown in this table, 4 patients had primary EGFR-negative tumors with EGFR-positive (1+) distant recurrences. Only 2 of these 4 patients received adjuvant chemotherapy. On the other hand, 2 patients with 2+ primary tumors had EGFR-negative distant recurrences and both patients received adjuvant chemotherapy. Also, 6 other patients had 1+ primary tumor but their recurrences were EGFR-negative. Only 4 of these 6 patients had adjuvant chemotherapy. Accordingly, in patients who received chemotherapy, the switch from positive to negative occurred in 6 patients and vice versa in 2 cases. To assess conformity of the two types of lesions, the weighted Kappa statistics produced a value of 0.44 representing a moderate degree of agreement.

**Table 2:** Results of EGFR immunostaining

**Primary**	**Distant Recurrence**			**Total**
		
	**Negative**	**1+**	**2+**	
**Negative**	8	4	0	12
**1+**	6	5	1	12
**2+**	2	1	6	9
**Total**	16	10	7	33

Negative No staining above the background

1+ Immunopositivity in < 50% of cells

2+ Immunopositivity in > 50% of cells

Further comparison between immunostaining of the primary tumors and their corresponding distant recurrences showed that in the 15 patients who received no postoperative chemotherapy, EGFR immunopositivity was decreased in only 3 distant recurrences but remained the same or increased in 12. In the group of 18 patients who received postoperative chemotherapy, EGFR immunostaining was decreased in 6 recurrences but remained the same or increased in 12. These results are summarized in Table 3. Although there was a higher tendency of distant recurrent tumors to show less EGFR immunostaining in the postoperative chemotherapy group, the difference between the two groups tested by Mantel-Haenszel Chi-square was not statistically significant (p = 0.6598). When comparing patients who did and did not receive adjuvant chemotherapy, those who received chemotherapy had an odds ratio of 4.75 (95% CI = 0.94 – 26.73) of having decreased EGFR immunostaining compared to those who did not receive chemotherapy.

**Table 3:** Postoperative chemotherapy and EGFR immunostaining.

Postoperative chemotherapy	**EGFR immunostaining of the distant recurrent tumor**			
	
	**Decreased**	**Same**	**Increased**	**Total**
**Yes**	6 (33.3%)	9 (50.0%)	3 (16.67%)	18
**No**	3 (20.0%)	10 (66.67%)	2 (13.33)	15
**Total**	9	19	5	33

Distant recurrence occurred 1 – 59 months (mean 14.9 ± 10.3) following the initial tumor resection. The difference between the median time-to-recurrence of primary tumors with the various degrees of staining was not statistically significant as shown by Log-Rank and Wilcoxon tests.

## Discussion

We assessed EGFR status of paired primary and distant recurrences of colorectal adenocarcinomas in a group of patients who received postoperative chemotherapy and compared their results with another group who did not receive any adjuvant therapy. We reported the results on a three-tier scale as negative (no staining), 1+ (positivity in <50% of cells) and 2+ (positivity in >50% of cells). We have reported earlier that the status of the primary tumor has a statistically significant predictive relationship to that of its recurrence when all tumors are collectively analyzed [10]. When the two groups were separated according to whether postoperative chemotherapy was administered, it was noted that recurrences following postoperative chemotherapy were approximately 5 times more likely to have lower levels of EGFR expression. These preliminary results document a trend which was not recognized earlier and may impact decision making when managing these patients with targeted biologic therapies especially since the anti-EGFR drug Cetuximab is only approved for patients who fail to respond to first line chemotherapy. The odds ratio for such recurrent tumors to exhibit lower levels of EGFR immunostaining compared to their originally resected primary is 4.75. The influence of postoperative chemotherapy on EGFR immunostaining in this small patient population was not statistically significant (p = 0.6598).

Several *in vitro *studies have shown that tumor cells which are sensitive to EGFR-targeted therapy, will also respond to the inhibitory effects of a number of cytotoxic drugs, which differ in their mechanism of action [14,15]. A recent study using colorectal cell lines showed that cells with high constitutive EGFR activity were not only sensitive to anti-EGFR agents but were also more likely to respond to oxaliplatin and 5-fluorouracil [16]. These findings may suggest that EGFR-positive cell clones within a colorectal adenocarcinoma may have an intrinsic susceptibility to postoperative chemotherapy due to complex and overlapping pathways. Consequently, a recurrent tumor may tend to include less EGFR-positive cells. The three-tier scale that we followed in recording our results allowed us to detect this trend, the statistical significance of which may need to be established in a larger series. Since EGFR-selective tyrosine kinase inhibitors are typically given to patients who have already been on chemotherapy, the tendency for a recurrent tumor to include less EGFR-positive cells especially if validated in larger series, could influence its sensitivity to this targeted therapy. A recent report on 16 patients with "EGFR-negative", chemotherapy-refractory tumors that responded to Cetuximab has been published [17]. At the current time, the selection or exclusion of patients for Cetuximab therapy on the basis of EGFR immunohistochemical testing remains a topic for further investigation.

In two previous studies of EGFR expression in colorectal carcinoma [9,18], the authors attempted reporting on the intensity of staining as weak, moderate and strong. However, their statistical analysis of data focused on the overall status of cases according to the percentage of positive cells irrespective of the intensity of staining. Therefore, in the current study we excluded the intensity of staining from the analysis and restricted our data collection to the percentage of positive cells. We believe that including the intensity of EGFR staining at this particular point in time while most pathology laboratories are still trying to agree on the methodology of reporting will add too much subjectivity into this process.

We used the antibody manufactured by Zymed Laboratories in the current study, which was one of the three antibodies equally recommended by The Canadian Consensus Panel on EGFR Testing in Colorectal Cancer. This panel is compromised of 12 practicing Canadian pathologists, who are recognized leaders in this field and holders of key positions at major Canadian Hospitals. They met in Toronto, Canada in September 2004 after reviewing the most recent practices in EGFR testing. The Panel agreed not to require the use of a specific antibody of EGFR testing, as there was no evidence in the medical literature to support the superiority of one antibody over another. The other two antibodies that were recommended in that meeting were manufactured by Dako (Mouse EGFR Clone H11 or DakoCytomation EGFR pharmDx kit) and Ventana (CONFIRM anti-EGFR [3C6] primary antibody) [13].

According to Atkins *et al*., [19], EGFR immunopositivity in colorectal cancer inversely correlates with the storage time of unstained slides. The authors recommended that specimens should be tested within the first 9 months to avoid false-negative results. The current is a retrospective study that specifically investigated whether adjuvant chemotherapy could influence the status of EGFR staining in recurrent colorectal cancer. Therefore, we included primary and recurrent tumor blocks for comparison with the understanding that storage might limit staining sensitivity. Our studied tissues were fixed in 10% neutral buffered formalin, which added stability of preservation and pH neutrality. They were stored for the average of 51 months.

## Conclusion

Our study shows that 57.6% of all recurrent colorectal adenocarcinomas will exhibit the same EGFR staining status of their primaries. However, patients who receive postoperative chemotherapy seem to be more likely to have recurrences with lower levels of EGFR immunostaining. Our results also showed that EGFR status could switch from positive to negative as well as from negative to positive with or without chemotherapy. Although the current report is limited by the small sample size, it brings up an observation that may stimulate further investigation, especially since EGFR-targeted drugs are typically given to patients who have already been on chemotherapy.

## Authors' contributions

**MAK**: Designed the project, collected cases, reviewed pathology and wrote most of the text.

**CHR**: Reviewed the literature, reviewed pathology, and wrote some of the text.

**RG**: Reviewed patients' charts, provided clinical data.

**YK**: Provided chemotherapy data, wrote some of the text.

**SH**: Performed most of recurrence surgeries, provided clinical information and wrote some of the text.

**AS**: Performed most of the primary surgeries, reviewed the literature and wrote some of the text.

**CL**: Performed some of the primary and recurrence surgeries, provided clinical information and wrote some of the text.

All authors read and approved the final manuscript.

## References

[B1] Salomon DS, Brandt R, Cardiello F, Normanno N (1995). Epidermal growth factor-related peptides and their receptors in human malignancies. Crit Rev Oncol Hematol.

[B2] Carpenter G, Cohen S (1990). Epidermal growth factor. J Biol Chem.

[B3] Real FX, Rettig WJ, Chesa PG, Melamed MR, Old LJ, Mendelsohn J (1986). Expression of epidermal growth factor receptor in human cultured cells and tissues: Relationship to cell lineage and stage of differentiation. Cancer Res.

[B4] Marmor MD, Skaria KB, Yarden Y (2004). Signal transduction and oncogenesis by ErbB/HER receptors. Int J Radiat Oncol Biol Phys.

[B5] Thomas SM, Grandis JR (2004). Pharmacokinetic and pharmacodynamic properties of EGFR inhibitors under clinical investigation. Cancer Treat Rev.

[B6] Saltz L, Hochster H, Rubin M (2001). Cetuximab (IMC-C225) plus irinotecan (CPT-11) is active in CPT-11-refractory colorectal cancer (CRC) that expresses epidermal growth factor receptor (EGFR) [abstract]. Proc Am Soc Clin Oncol.

[B7] Saltz L, Meropol N, Loehrer P, Needle M, Kopit J, Mayer R (2004). Phase II trial of cetuximab in patients with refractory colorectal cancer that expresses the epidermal growth factor receptor. J Clin Oncol.

[B8] Cunningham D, Humblet Y, Siena S, Khayat D, Bleiberg H, Santoro A, Bets D, Mueser M, Harstrick A, Verslype C, Chau I, Van Cutsem E (2004). Cetuximab monotherapy and cetuximab plus irinotecan in irinotecan-refractory metastatic colorectal cancer. New Engl J Med.

[B9] Italiano A, Saint-Paul MC, Caroli-Bosc FX, François E, Bourgeon A, Benchimol D, Gugenheim J, Michiels J-F (2005). Epidermal growth factor receptor (EGFR) status in primary colorectal tumors correlates with EGFR expression in related metastatic sites: biological and clinical implications. Ann Oncol.

[B10] Khalifa MA, Rowsell CH, Gladdy RA, Ko Y-J, Hanna S, Smith A, Law C (2006). Expression of the Epidermal Growth Factor Receptor (EGFR) in Primary Colorectal Adenocarcinoma Predicts its Expression in Recurrent Disease. Am J Clin Pathol.

[B11] Bibeau F, Boissière-Michot F, Sabourin J-C, Gourgou-Bourgade S, Radal M, Penault-Llorca F, Rochaix P, Arnould L, Barlet M-P, Azria D, Ychou M (2006). Assessment of epidermal growth factor receptor (EGFR) expression in primary colorectal carcinomas and their related metastases on tissue sections and tissue microarray. Virchow Arch.

[B12] De Pas T, Pelosi G, de Braud F, Veronesi G, Curigliano G, Leon ME, Danesi R, Noberasco C, d'Aiuto M, Catalano G, Viale G, Spaggiari L (2004). Modulation of epidermal growth factor receptor status by chemotherapy in patients with locally advanced non-small-cell lung cancer is rare. J Clin Oncol.

[B13] Banerjee D, Guba AK, Guindi MM, Haliotis T, Hanna W, Jass JR, Jothy S, Kanthan R, O'Malley F, Srigley J, Têtu B, Wightman R, Dranitsaris G (2005). Best practice standards for EGFR testing in colorectal cancer in Canada.

[B14] Ciardiello F, Caputo R, Bianco R, Damiano V, Pomatico G, DePlacido S, Bianco AR, Tortora G (2000). Antitumor effect and potentiation of cytotoxic drugs activity in human cancer cells by ZD-1839 (Iressa), an epidermal growth factor receptor-selective tyrosine kinase inhibitor. Clin Cancer Res.

[B15] Sirotnak FM, Zakowski MF, Miller VA, Scher HI, Kris MG (2000). Efficacy of cytotoxic agents against human tumor xenografts is markedly enhanced by coadministration of ZD1839 (Iressa), an inhibitor of EGFR tyrosine kinase. Clin Cancer Res.

[B16] Van Schaeybroeck S, Karaiskou-McCaul A, Kelly D, Longley D, Galligan L, Van Cutsem E, Johnston P (2005). Epidermal growth factor receptor activity determines response of colorectal cancer cells to Gefitinib alone and in combination with chemotherapy. Clin Cancer Res.

[B17] Chung KY, Shia J, Kemeny NE, Shah M, Schwartz GK, Tse A, Hamilton A, Pan D, Schrag D, Schwartz L, Klimstra DS, Fridman D, Kelsen DP, Saltz LB (2005). Cetuximab shows activity in colorectal cancer patients with tumors that do not express the epidermal growth factor receptor by immunohistochemistry. J Clin Oncol.

[B18] Scartozzi M, Bearzi I, Berardi R, Mandolesi A, Fabris G, Cascinu S (2004). Epidermal growth factor receptor (EGFR) status in primary colorectal tumors does not correlate with EGFR expression in related metastatic sites: Implications for treatment with EGFR-targeted monoclonal antibodies. J Clin Oncol.

[B19] Atkins D, Reiffen KA, Tegtmeier CL, Winther H, Bonato MS, Strokel S (2004). Immunohistochemical detection of EGFR in paraffin-embedded tumor tissues: variation in staining intensity due to choice of fixative and storage time of tissue sections. J Histochem Cytochem.

